# Toxicity of spray adjuvants and tank mix combinations used in almond orchards to adult honey bees (*Apis mellifera*)

**DOI:** 10.1093/jee/toad161

**Published:** 2023-09-01

**Authors:** Brandon Shannon, Emily Walker, Reed M Johnson

**Affiliations:** Department of Entomology, The Ohio State University, 1680 Madison Avenue, Wooster, OH, USA; Department of Entomology, The Ohio State University, 1680 Madison Avenue, Wooster, OH, USA; Department of Entomology, The Ohio State University, 1680 Madison Avenue, Wooster, OH, USA

**Keywords:** adjuvant, honey bee, pesticide, toxicity, surfactant

## Abstract

Commercial beekeepers transporting honey bees across the United States to provide almond pollination services have reported honey bee deaths, possibly due to pesticide applications made during crop bloom. Pesticides are often applied as “tank mixes”, or mixtures of fungicides and insecticides combined into a single application. Spray adjuvants are often added to tank mixes to improve the application characteristics of a pesticide and include spreaders, stickers, or surfactants. The goal of this research was to determine toxicity of adjuvants to adult worker honey bees, both when applied alone and in adjuvant-pesticide tank mixtures. Field-relevant combinations of formulated products were applied to 3-day-old adult worker honey bees using a Potter Spray Tower, and mortality was assessed 48 h following exposure. Adjuvants tested included Activator-90, Attach, Choice Weather Master, Cohere, Dyne-Amic, Induce, Kinetic, LI 700, Liberate, Nu-Film P, PHT Latron B-1956, and Surf-90; fungicides tested include Luna Sensation (Fluopyram and Trifloxystrobin), Pristine (Pyraclostrobin and Boscalid), Tilt (Propiconazole), and Vangard (Cyprodinil), and insecticides tested include Altacor (Chlorantraniliprole), Intrepid 2F (Methoxyfenozide), and a positive control Mustang Maxx (Zeta-cypermethrin). Results demonstrated that exposure to some adjuvants causes acute honey bee mortality at near-field application rates, both when applied alone and in combination with pesticides. Some adjuvant-pesticide combinations demonstrated increased toxicity compared with the adjuvant alone, while others demonstrated decreased toxicity. A better understanding of adjuvant and adjuvant-pesticide tank mixture toxicity to honey bees will play a key role in informing “Best Management Practices” for pesticide applicators using spray adjuvants during bloom when honey bee exposure is likely.

## Introduction

Honey bees are responsible for pollination of over 100 commercial crops in North America (Hristov et al. 2020), with an estimated contribution of 12 billion USD to the US economy (Calderone 2012, Khalifa et al. 2021). Due to the enormous mass-flowering of almonds in California, commercial beekeepers transport bees across the United States to satisfy the pollination needs of this crop (Goodrich et al. 2019). In 2021, approximately 500,000 ha of almonds required an estimated 2.6 million honey bee colonies for pollination services, which makes up 90% of the 2.92 million managed honey bee colonies in the United States (Goodrich et al. 2021).

**Fig. 1.**
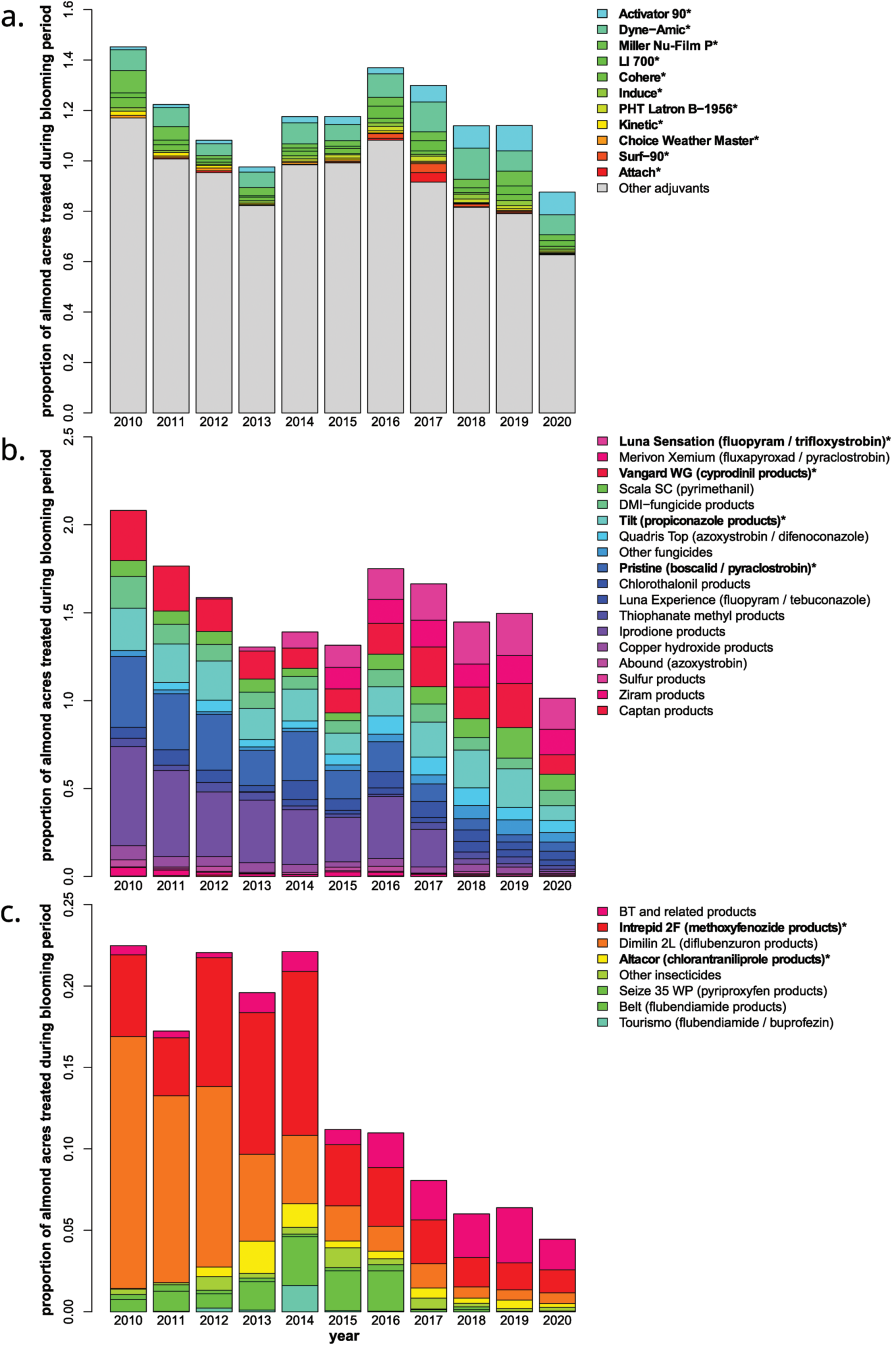
Usage data for adjuvants (a), fungicides (b), and insecticides (c) applied to almond orchards, expressed in proportion of almond acres treated during the blooming period (15 February–15 March). The height of each box represents the proportion of almond-bearing acres receiving that treatment. The legends in each graph correspond to each treatment. Products with an asterisk were used in combination toxicity testing for this study. Data were summarized from pesticide usage reporting in the California Pesticide Information Portal (State of California – California Pesticide Information Portal 2023).

A 2021 survey found that 19% of beekeepers providing bees for almond pollination observed lethal effects and 56% observed sublethal effects in their colonies that they attributed to pesticide exposure (Goodrich et al. 2021). Almond growers often apply a variety of agrochemicals during bloom to which honey bees may be exposed (State of California – California Pesticide Information Portal 2023). These applications may contain multiple agrochemicals combined into a “tank mix” and usually include a fungicide, sometimes include an insecticide, and often include one or more spray adjuvants that are intended to improve spray performance (Mullin et al. 2015, Durant et al. 2020). Insecticides applied to almonds during bloom (Fig. 1c) are used with the intent of controlling insect pests such as the peach twig borer (*Anarsia lineatella*) (Zalom et al. 2017). Fungicides are applied to control fungal diseases of almonds (Fig. 1b) including brown rot (*Monilinia laxa*) and anthracnose (*Colletotrichum acutatum*) (Doll 2012). Since fungicides do not target insects, their effects on honey bees are not as clearly established. However, studies have shown that fungicides can affect the behavior, metabolism, immune response, and gut microbiota in bees, in addition to causing histopathological effects in the midgut (Cizelj et al. 2016, Liao et al. 2017, Mao et al. 2017, Batista et al. 2020, Al Naggar et al. 2022).

There are federal laws for bee hazard labeling, which are intended to mitigate the impacts of pesticide application on bees (US EPA 2013b). The state of California has additional laws that prevent pesticides that are harmful to bees from being applied in daytime hours during bloom (State of California – California Code of Regulations 2022). These restrictions are informed by toxicity testing for nontarget organisms (US EPA 2013a). However, this testing is generally performed only on individual active ingredients rather than field-relevant combinations of formulated products. Pesticides applied to almonds during bloom generally have no bee hazard labeling that would indicate honey bee toxicity when applied alone.

Pesticides, however, are not always applied alone. Combined mixtures of particular insecticides and fungicides have demonstrated synergistic toxicity to honey bees (Pilling et al. 1995, Vandame and Belzunces 1998, Thompson and Wilkins 2003, Biddinger et al. 2013, Johnson and Percel 2013, Johnson et al. 2013, Zhu et al. 2014, Wade et al. 2019, Walker et al. 2022). Pesticide tank mixes may also include spray adjuvants, products intended to improve the application properties of the pesticide mixture. Spray adjuvants can have many functions, including adjustment of pH, antifoaming, drift reduction, enhanced sticking, and reduction of surface tension through surfactant activity to increase wetting, spreading, and penetration (Young and Matthews 2013). A wide variety of adjuvant products are used during almond bloom, shown in Fig. 1a (State of California – California Pesticide Information Portal 2023).

The ingredients that make up formulated adjuvants are called “principal functioning agents” (Council of Producers and Distributors of Agrotechnology 2020). In contrast to traditional pesticides, adjuvant labels and marketing materials do not claim any pesticidal activity, and the principal functioning agent constituents in adjuvants are considered “inert ingredients” and do not undergo the same testing and risk assessment that is required of pesticides (US EPA 2013c). The main classes of principal functioning agents with surfactant properties are (i) nonionic surfactants, (ii) crop oil concentrates, (iii) modified seed oils, (iv) organo-silicone surfactants, and (v) hydrocolloid polymers. These can be formulated alone or in combination with other adjuvant classes (Abbott et al. 2021). Although no pesticidal activity is claimed, some adjuvants have been noted to demonstrate toxicity to pest arthropods (Searle et al. 1990, Cloyd 2005). Moreover, a study from New Zealand showed that formulated surfactants can cause honey bee mortality at field-relevant rates when applied as a spray (Goodwin and McBrydie 2000). However, with the vast quantity and diversity of adjuvant products, more testing is needed to assess the risk of formulated adjuvants commonly used during bloom to honey bees.

Organo-silicones are one class of adjuvant “principal functioning agent” that have raised concerns in regard to bee toxicity (Mullin 2015). Organo-silicones decrease surface tension and increase spreading on waxy surfaces, even when used at rates as low as 10% of that of traditional surfactants. Organo-silicone surfactants can be ring structured, but most have a chain structure with a dimethylsiloxane polymer attached to an ether formed from ethylene oxide terminated with an organic reactive group. The dimethylsiloxane structure can be linear or branched and the organic reactive group can vary with corresponding changes in chemical and physical properties (Adams 1991). Most commonly 3 dimethylsiloxane units form a trisiloxane head, but other polymeric dimethylsiloxanes are also common. Oral exposure to trisiloxane organo-silicone surfactants can impair honey bee learning (Ciarlo et al. 2012, Chen et al. 2018), is acutely toxic to adult honey bees, and can increase the pathogenicity of bee viruses (Fine et al. 2017). Chronic exposure to organo-silicones has been shown to cause mortality in honey bee larvae (Mullin 2015, Fine et al. 2017). Combinations of organo-silicone adjuvants with insecticides, fungicides, and herbicides also show synergistic toxicity to honey bees (Mullin et al. 2015, Walker et al. 2022, Wernecke et al. 2022). Trisiloxane surfactants are not commonly found in air, rapidly degrade in water, and do not readily travel from plant roots to leaves and flowers. Therefore, exposure to trisiloxane surfactants is only known to occur in honey bees through direct spraying of foraging bees and contact with pollen that has been sprayed directly (Slade 2020).

A type of nonionic surfactant, organic ethoxylates, is another class of adjuvant “principal functioning agents” that have received attention regarding bee toxicity. Organic ethoxylates encompass alcohol ethoxylates, fatty acid ethoxylates, and amine ethoxylates and are made up of an alkyl or aryl alcohol, fatty acid, or amine bonded to an ether chain formed from ethylene oxide. The alkyl or aryl group can be varied and the molar ratio of ethylene oxide added can be modified to change the physical and chemical properties. Organic ethoxylates are typically classified as nonionic surfactants, though some amine ethoxylates act as cationic surfactants due to the positively charged amine group (Rapp 2017). Nonylphenol ethoxylate can impair honey bee learning (Mullin 2015). Tergitol, a commercially available alcohol ethoxylate, is used as a mosquito larvicide that reduces water tension and causes larval drowning (Ware and Whitacre 2004).

The other classes of adjuvant principal functioning agents have received less attention with regards to honey bee toxicity. However, N-methyl-2-pyrrolidone, an emulsifying agent, is moderately toxic to *Daphnia magna*, honey bee larvae, and adult honey bees (Lan et al. 2004, Mullin 2015, Fine and Mullin 2017). Most adjuvant toxicity studies focus on a single surfactant compound, while many popular adjuvant products contain multiple “principal functioning agents” from different classes. Additionally, there is little published information on the effect of adjuvants combined with insecticides or fungicides despite the fact that adjuvants are always applied in combination with a pesticide and never alone.

This study builds on a previous finding that mixtures of particular insecticides and fungicides applied to almonds during bloom can cause elevated mortality in adult worker bees when mixed with the spray adjuvant Dyne-Amic (Walker et al. 2022). To determine whether other adjuvant products also have the potential to increase the toxicity of pesticides to bees, 6 adjuvants in addition to Dyne-Amic were tested in field-relevant tank mix combinations. The intrinsic toxicity of 11 adjuvants in addition to Dyne-Amic was determined in the absence of pesticides. This study simulated direct spray exposure to adjuvants and pesticides using a Potter Spray Tower (Potter 1952) to determine the acute lethal effects on 3-day-old adult worker honey bees. Twelve formulated adjuvants, 4 formulated fungicides, and 2 formulated insecticides commonly used in almond orchards during bloom were sprayed on bees at multiples of the maximum labeled rate to allow for the direct comparison of results to application scenarios in the field.

## Materials and Methods

### Test Chemicals

This study used 4 fungicide formulations and 2 insecticide formulations commonly used in California during almond bloom (Table 1). The insecticide Mustang Maxx (active ingredient zeta-cypermethrin), a pyrethroid insecticide with high acute toxicity to honey bees that is not applied to almonds during bloom, was used as a positive control (Zhu et al. 2014, Fanning et al. 2018, Walker et al. 2022). The pesticides chosen contain different active ingredients and work through different modes of action. Twelve adjuvant products commonly used in California during almond bloom were tested (Table 2). The adjuvants Activator-90, Attach, Choice Weather Master, PHT Latron B-1956, and Liberate were not tested in combination with pesticides due to their demonstrated low toxicity to bees when tested alone. The insecticide Altacor was not tested in combination with all adjuvants. Adjuvants tested include a range of constituent principal functioning agents that function as wetters, spreaders, penetrants, surfactants, or water conditioning agents. The labeled usage rates vary between adjuvants, with maximum labeled application rates ranging from 8 to 80 fl. oz. per 100 gallons.

**Table 1.** Label information on pesticides tested. No pesticide labels included language prohibiting application around honey bees, except Mustang Maxx, which was included as a positive control. The percentage by weight of each active ingredient is as stated on the pesticide label. The maximum recommended application rate on almonds is represented with each active ingredient individually as pounds of active ingredient per acre and as the sum of all active ingredients as milligrams of total active ingredients per liter

Pesticide	Active ingredient (%)	Mode of action	Manufacturer	Revision date	Maximum application rate		
					fl oz./A or oz./A	lb. AI/A	mg total AI/L
Altacor Insecticide	Chlorantraniliprole (35%)	IRAC Group 28	FMC Corporation	2 July 2018	4.5 fl. oz./A	0.099	73.2
Intrepid 2F Insecticide	Methoxyfenozide (22.6%)	IRAC Group 18	Corteva Agriscience, LLC	5 July 2017	24 fl. oz./A	0.38	267
Luna Sensation Fungicide	Fluopyram (21.4%), Trifloxystrobin (21.4%)	FRAC Groups 7 and 11	Bayer CropScience LP	12 June 2019	7.6 fl. oz./A	0.125, 0.125	177
Pristine Fungicide	Pyraclostrobin (12.8%), Boscalid (25.2%)	FRAC Groups 7 and 11	BASF Corporation	30 October 2018	14.5 oz./A	0.116, 0.228	245
Tilt Fungicide	Propiconazole (41.8%)	FRAC Group 3	Syngenta Crop Protection, LLC	6 September 2019	8 fl. oz./A	0.22	160
Vangard WG Fungicide	Cyprodinil (75%)	FRAC Group 9	Syngenta Crop Protection, LLC	26 March 2020	10 oz./A	0.469	334
Mustang Maxx Insecticide (Positive Control)	Zeta-cypermethrin (9.15%)	IRAC Group 3A	FMC Corporation	4 June 2019	4 fl. oz./A	0.025	17.8

**Table 2.** Label information for adjuvant products tested. No adjuvant label included language prohibiting application around honey bees. The maximum recommended application rate on almonds is listed on the adjuvant label as fl. oz. of adjuvant formulation per 100 gallons

Adjuvant	Manufacturer	Registration date	Max app. rate		Principal functioning agents	Adjuvant function
			fl oz./100 gal.	mg/L		
Activator-90	Loveland Products, Inc.	9 July 2013	64	5,050	Alkylphenol ethoxylate, alcohol ethoxylate, and tall oil fatty acid	Nonionic surfactant, penetrant, antifoaming agent
Attach	Loveland Products, Inc.	29 January 2004	16	1,156	Pinene (terpene) polymers, petrolatum, a-(p-Dodecylphenyl)-Omega-hydroxypoly (oxyethylene)	Spreader, sticker
Choice Weather Master	Loveland Products, Inc.	30 November 2004	64	5,900	Propionic acid ammonium salt, alkylphenol ethoxylate phosphate ester, 2-hydroxy-1,2,3-propanetricarboxylic acid, ammonium sulfate, acrylic acid polymer sodium salt	Water conditioning agent
Cohere	Helena Agri-Enterprises, LLC	26 August 1999	16	1,275	Alkanolamide surfactants, alkylaryl polyethoxyethanol sulfates, 1,2-propanediol	Nonionic spreader-sticker
Dyne-Amic	Helena Agri-Enterprises, LLC	3 December 1992	80	5,719	Methyl esters of C16-C18 fatty acids, polyalkyleneoxide modified polydimethylsiloxane, alkylphenol ethoxylate	Methylated seed oil, organo-silicone surfactant
Induce	Helena Agri-Enterprises, LLC	18 April 2002	48	3,729	Alkyl aryl polyoxylkane ethers, alkanolamides, dimethyl siloxane, free fatty acids	Nonionic low foam wetter/spreader
Kinetic	Helena Agri-Enterprises, LLC	8 May 2001	64	5,125	Polyalkyleneoxide modified polydimethylsiloxane and nonionic surfactants	Nonionic wetter/spreader/penetrant
LI 700	Loveland Products, Inc.	13 July 2004	32	646	Phosphatidylcholine, methylacetic acid, alkyl polyoxyethylene ether	Penetrant, acidifier, deposition aid, drift control agent
Liberate	Loveland Products, Inc.	3 June 2004	32	2,600	Lecithin, methyl esters of fatty acids, alcohol ethoxylate	Penetrant, deposition aid, drift control agent
Nu-Film P	Miller Chemical and Fertilizer, LLC	14 April 2015	16	2,450	Pinene (polyterpenes) polymers, petrolatum, alkyl amine ethoxylate	Spreader, sticker
PHT Latron B-1956	J. R. Simplot Company	25 October 2012	8	1,156	Modified phthalic glycerol alkyd resin	Spreader, sticker
Surf-90	Mar Vista Resources	13 July 2004	64	5,000	Alkylphenol ethoxylate, polyethylene glycol, tall oil fatty acids	Wetter, spreader, nonionic, surfactant

### Honey Bees

The methods for collection of honey bees in this study meet guidelines stated in the US EPA OCSPP 850.3020: Honey Bee Acute Contact Toxicity Test, which specifies that tests must be conducted on workers that are of a similar age and feeding status (US EPA 2012). Frames of late-stage capped worker brood were collected from healthy honey bee colonies maintained at the Ohio State University Wooster campus that were managed according to beekeeping best management practices (Kulhanek et al. 2021). Colonies were requeened annually with New World Carniolan queens (*Apis mellifera carnica*). No antibiotics were used to control bacterial diseases in the 5 yr prior to collection and only formic acid and oxalic acid were used for control of *Varroa destructor*, as needed, and at least 1 month prior to experiments. Brood frames were collected and placed in a closed plastic nucleus colony box (Bee Brief, Nod Apiary Products, Frankford, Ontario, Canada) and stored in an incubator under hive conditions (darkness, 34 °C, 60–80% relative humidity; incubator Model HH030-AA, Darwin Chambers, St. Louis, MO, USA). Frames were collected every 3–7 days from a minimum of 3 different colonies for each treatment, out of at least 8 different source colonies providing bees for all experiments conducted during May–September 2018–2021. Bees that emerged within the previous 24 h were brushed from frames and stored in the incubator inside wooden cages (11 × 14 × 22 cm). Bees with signs of disease, such as deformed wings, or with visible *Varroa* mites were discarded. Cages were stored in the incubator for 72 hr prior to spray application and provisioned with 1:1 (w/w) sucrose water solution, prepared no more than 1 wk prior to feeding and stored in darkness at 4 °C.

### Preparation for Acute Toxicity Tests

Spray treatments were applied at multiples of the maximum labeled field application rate for almonds, scaled to the area of a 9-cm-diameter circular petri dish. Pesticides and adjuvants tested in this study are referred to only by their product name. Determination of application rates was performed following procedures outlined in Walker et al. (2022). Adjuvant application rates were determined using concentrations of the 1-ml Potter Tower spray volume, which is equivalent to approximately 1,572 liters per ha (168 gallons per acre) when applied to the area of a 9-cm petri dish. A concentrated stock solution (100 times the field application rate) of each pesticide was diluted with distilled water at room temperature to make the desired multiple of the maximum field application rate (0.1–100×). When adjuvant-pesticide combinations were tested, adjuvants were directly pipetted from stock into these solutions, so that both pesticide and adjuvant were the same multiple of the field application rate. When an adjuvant alone was tested, adjuvants were directly pipetted from stock into distilled water. All solutions were stored at 4 °C in darkness.

### Acute Toxicity Tests With the Potter Spray Tower

Cages of 3-day-old honey bees were first anesthetized for 3 min with carbon dioxide. Anesthetized bees were separated into groups of 20 in plastic-coated paper cups (490 ml, UNIQ 8 oz Cups, Frozen Dessert Supplies, Rexburg, ID, USA), covered with #20 cotton cheesecloth secured with a #32 rubber band. For spray treatment, each group of bees were again anesthetized with carbon dioxide for 15–20 s and transferred to a 9-cm glass petri dish covered with 9-cm filter paper (Whatman qualitative filter paper, Grade 1, 90 mm circles) and placed onto the spray plate of the Potter Spray Tower. Air pressure for the Potter Spray Tower was set to 68.9 kPa (10 psi) for all treatments. Bees were then sprayed with 1 ml of the designated treatment, returned to the labeled cup, and fed with a double-punctured 1.5-m microcentrifuge tube filled with sucrose solution (1:1 w/w). Bees were sprayed in treatments starting with DI water as the negative control, then proceeding from lowest to highest concentration, where a minimum of 4 concentrations and a negative control constituted a full series of treatments in a group. Concentrations varied between treatments, but the respective concentrations used for each treatment are listed in Tables 3 and 4. The number of replicates varied between treatments as additional concentrations were added after initial bioassays to increase precision for LD_50_ and LC_50_ estimates. Each treatment had a minimum of 3 treatment series replicates and a minimum of 4 treatment concentrations, for a minimum of 240 bees tested (number of bees). Treatment groups with control mortality exceeding 10% were discarded and excluded from analysis following the Ecological Effects Test Guidelines outlined in OCSPP 850.3020 (US EPA 2012). Mustang Maxx was applied at the field application rate as a positive control. The Potter Tower was cleaned with water and acetone between each treatment series. Bees were returned to the incubator and the number of dead bees was recorded at 48 h, which is the timeframe used for acute toxicity in the Ecological Effects Test Guidelines outlined in OCSPP 850.3020 (US EPA 2012).

**Table 3.** Adjuvants, pesticides, and adjuvant-pesticide combinations determined to have a dose–response curve not significantly different from zero (*P*-value > 0.05) using the noEffect function in the drc package (Ritz et al. 2015).

Treatment	Type	Concentrations tested	Number of bees
		*X* application rate	
Altacor alone	Insecticide	1, 3, 10, 30, 50	600
Intrepid 2F alone	Insecticide	0.1, 1, 3, 10, 20, 30, 40, 50, 100	1,597
Luna Sensation alone^a^	Fungicide	1, 3, 10, 30, 50, 100	1,582
Pristine alone	Fungicide	0.1, 1, 3, 10, 30, 50	1,211
Tilt alone^a^	Fungicide	0.1, 1, 3, 10, 30, 50, 100	2,221
Vangard alone^a^	Fungicide	1, 3, 5, 8, 10, 15, 20, 30, 50, 100	1,451
Attach Alone	Adjuvant	1, 3, 10, 30	240
Dyne-Amic + Tilt^a^	Adjuvant	0.1, 1, 3, 5, 8, 10, 15, 30	958
LatronB alone^a^	Adjuvant	1, 3, 5, 8, 10, 15, 30, 50	756
Nu-Film P alone	Adjuvant	1, 3, 10, 30	677
Nu-Film P + Altacor	Adjuvant + Insecticide	1, 3, 10, 30	180
Nu-Film P + Intrepid 2F	Adjuvant + Insecticide	1, 3, 10, 30	240
Nu-Film P + Luna Sensation	Adjuvant + Fungicide	1, 3, 10	237
Nu-Film P + Pristine	Adjuvant + Fungicide	1, 3, 10, 30	539
Nu-Film P + Tilt^a^	Adjuvant + Fungicide	1, 3, 10, 30	238
Nu-Film P + Vangard	Adjuvant + Fungicide	1, 3, 10, 30	239

^a^Treatments with a significant dose–response relationship but with an LC_20_ value greater than the maximum concentration tested are also included.

**Table 4.** LC_50_ and LD_50_ estimates for adjuvants alone, adjuvant-pesticide tank combinations, and Mustang Maxx insecticide positive control that demonstrated a significant dose–response relationship. LC_50_ estimates represent the application rate of all components in the treatment mixture. LD_50_ estimates represent the dose per bee of each adjuvant and pesticide active ingredient

Treatment	Type	Number of bees	Concentrations tested	Treatment LC_50_ estimate	Adjuvant LD_50_ estimate	Pesticide LD_50_ Estimate	Slope
			*X* Application rate	*X* Application rate	µg/bee	µg total A.I./bee	−*b*
Kinetic alone	Adjuvant	1,120	0.1, 0.3, 1, 3, 5, 8, 10, 15, 20, 30, 50	8.8 (8.1–9.4)	18.4 (17.0–19.7)	N/A	2.4 (2.1–2.8)
Kinetic + Intrepid 2F	Adjuvant + Insecticide	365	1, 3, 5, 8, 10, 15	3.6*** (3.0–4.2)	10.0*** (7.9–12.1)	0.395 (0.327–0.463)	1.6 (1.2–1.9)
Kinetic + Luna Sensation	Adjuvant + Fungicide	410	1, 3, 5, 8, 10, 15	3.6*** (2.9–4.3)	10.8*** (9.6–12.1)	0.259 (0.208–0.311)	1.3 (1.0–1.6)
Kinetic + Pristine	Adjuvant + Fungicide	565	1, 3, 5, 8, 10, 15, 20, 30	4.8*** (3.8–5.8)	7.5*** (6.3 –8.8)	0.478 (0.376–0.579)	0.90 (0.70–1.1)
Kinetic + Tilt	Adjuvant + Fungicide	485	1, 3, 5, 8, 10, 15	5.2*** (4.6–5.8)	7.5*** (6.0–9.0)	0.339 (0.301–0.378)	2.1 (1.7 -2.4)
Kinetic + Vangard	Adjuvant + Fungicide	404	1, 3, 5, 8, 10, 15, 20, 30	3.6*** (3.0–4.2)	7.6*** (6.3–8.9)	0.492 (0.412–0.573)	1.8 (1.5–2.2)
Surf-90 alone	Adjuvant	1,263	0.3, 1, 1.3, 3, 5, 8, 10, 12, 15, 20, 30	8.9 (8.3–9.6)	18.2 (16.9–19.5)	N/A	1.9 (1.7–2.2)
Surf-90 + Intrepid 2F	Adjuvant + Insecticide	401	0.3, 1, 3, 5, 8, 10, 15	7.3** (6.5–8.1)	14.8** (13.2–16.5)	0.793 (0.705–0.881)	2.4 (1.9–2.9)
Surf-90 + Luna Sensation	Adjuvant + Fungicide	551	0.3, 1, 1.3, 3, 5, 8, 10, 15	7.3** (6.5–8.1)	14.9** (13.3–16.6)	0.529 (0.470–0.588)	1.9 (1.6–2.3)
Surf-90 + Pristine	Adjuvant + Fungicide	471	0.3, 1, 1.3, 3, 5, 8, 10, 15	8.4 (7.5–9.2)	17.2 (15.4–18.9)	0.842 (0.758–0.927)	2.6 (2.1–3.1)
Surf-90 + Tilt	Adjuvant + Fungicide	610	0.3, 1, 1.3, 3, 5, 8, 10, 15	6.6*** (6.0–7.3)	13.5*** (12.2–14.9)	0.433 (0.390–0.476)	2.1 (1.8–2.5)
Surf-90 + Vangard	Adjuvant + Fungicide	495	0.3, 1, 1.3, 3, 5, 8, 10, 15	4.4*** (3.9–4.9)	9.0*** (7.9–10.0)	0.599 (0.526–0.671)	2.0 (1.7–2.4)
Induce alone	Adjuvant	402	1, 3, 10, 15, 20, 30, 50	10.8 (9.3–12.2)	16.4 (14.1–18.7)	N/A	2.2 (1.7–2.6)
Induce + Intrepid 2F	Adjuvant + Insecticide	547	1, 3, 5, 8, 10, 15, 20, 30	6.7*** (6.0–7.5)	10.2*** (9.1–10.3)	0.735 (0.655–0.814)	2.1 (1.8–2.5)
Induce + Luna Sensation	Adjuvant + Fungicide	469	1, 3, 5, 8, 10, 15, 20, 30	9.0 (8.0–10.1)	18.4 (17.0–19.7)	0.652 (0.577–0.727)	2.0 (1.7–2.4)
Induce + Pristine	Adjuvant + Fungicide	423	1, 3, 5, 8, 10, 15, 20, 30	6.7*** (6.0–7.4)	10.0*** (7.9–12.1)	0.671 (0.600–0.742)	2.6 (2.1–3.0)
Induce + Tilt	Adjuvant + Fungicide	347	1, 3, 5, 8, 10, 15, 20	7.1*** (6.1–8.0)	10.8*** (9.2–12.3)	0.462 (0.397–0.527)	1.8 (1.4–2.2)
Induce + Vangard	Adjuvant + Fungicide	479	1, 3, 5, 8, 10, 15, 20, 30	6.0*** (5.4–6.6)	9.2*** (8.2–10.1)	0.823 (0.738–0.908)	2.8 (2.3–3.3)
Dyne-Amic alone	Adjuvant	1,995	0.1, 1, 3, 5, 8, 10, 15, 20, 30, 50	13.4 (12.0–14.8)	31.3 (28.1–34.5)	N/A	1.1 (1.0–1.2)
Dyne-Amic + Altacor	Adjuvant + Insecticide	600	1, 3, 5, 8, 10, 15, 20, 30	17.1*** (15.7–18.4)	39.9*** (36.7–43.1)	0.511 (0.470–0.551)	3.4 (2.9–4.0)
Dyne-Amic + Intrepid 2F	Adjuvant + Insecticide	763	1, 3, 5, 8, 10, 15, 20, 30	29.7*** (24.7–34.7)	69.4*** (57.6–81.1)	3.24 (2.69–3.79)	1.8 (1.4–2.1)
Dyne-Amic + Luna Sensation	Adjuvant + Fungicide	1,019	1, 3, 5, 8, 10, 15, 20, 30	23.8*** (20.6–27.0)	55.6*** (48.2–63.0)	1.72 (1.49–1.95)	1.6 (1.4–1.9)
Dyne-Amic + Pristine	Adjuvant + Fungicide	820	1, 3, 5, 8, 10, 15, 20, 30	15.4* (14.2–16.7)	36.1* (33.2–39.0)	1.55 (1.42–1.67)	2.7 (2.3–3.1)
Dyne-Amic + Vangard	Adjuvant + Fungicide	996	1, 3, 5, 8, 10, 15, 20, 30	68.4*** (34.9–102)	159.9*** (81.5–238.3)	9.34 (4.76–13.9)	0.75 (0.57–0.93)
Cohere alone	Adjuvant	463	0.595, 1, 1.785, 3, 5.95, 10, 15, 17.85, 20, 30, 50	24.8 (20.5–29.1)	12.9 (10.7–15.2)	N/A	1.4 (1.1–1.7)
Cohere + Intrepid 2F	Adjuvant + Insecticide	404	1, 3, 5, 8, 10, 15, 20, 30	99.1*** (6.0–192)	51.6*** (3.1–100.2)	10.8 (0.650–21.0)	1.1 (0.57–1.6)
Cohere + Luna Sensation	Adjuvant + Fungicide	462	1, 3, 5, 8, 10, 15, 20, 30	51.5*** (26.8–76.1)	26.8*** (14.0–39.7)	3.72 (1.94–5.51)	1.3 (0.86–1.8)
Cohere + Pristine	Adjuvant + Fungicide	528	0.595, 1, 1.785, 3, 5.95, 8, 10, 15, 17.85, 20, 30	24.7 (20.3–29.2)	12.9 (10.6–15.2)	2.48 (2.03–2.93)	2.0 (1.5–2.5)
Cohere + Tilt	Adjuvant + Fungicide	500	0.595, 1, 1.785, 3, 5.95, 8, 10, 15, 17.85, 20, 30	21.4 (17.1–25.8)	11.2 (8.9–13.4)	1.40 (1.12–1.69)	1.4 (1.1–1.8)
Cohere + Vangard	Adjuvant + Fungicide	345	1, 3, 5, 8, 10, 15, 20, 30	22.3 (19.7–25.0)	11.6 (10.2–13.0)	3.05 (2.68–3.42)	3.1 (2.3–3.9)
LI 700 alone	Adjuvant	1,475	1, 3, 5, 8, 10, 15, 30, 40, 50	90.6 (59.8–122)	96.3 (63.6–129.1)	N/A	0.79 (0.65–0.94)
LI 700 + Altacor	Adjuvant + Insecticide	440	1, 3, 10, 30, 40	97.4 (38.4–157)	103.5 (40.8–166.3)	2.92 (1.15–4.68)	1.2 (0.75–1.7)
LI 700 + Intrepid 2F	Adjuvant + Insecticide	520	1, 3, 10, 30, 40, 50	128* (49.6–206)	135.6* (52.7–218.5)	13.9 (5.42–22.5)	1.2 (0.73–1.6)
LI 700 + Luna Sensation	Adjuvant + Fungicide	520	1, 3, 10, 30, 40, 50	192*** (30.3–354)	204.2*** (32.2–376.1)	13.9 (2.19–25.6)	0.87 (0.54–1.2)
LI 700 + Pristine	Adjuvant + Fungicide	522	1, 3, 10, 30, 40, 50	41.3*** (35.9–46.8)	43.9*** (38.1–49.8)	4.15 (3.60–4.70)	2.2 (1.6–2.7)
LI 700 + Tilt	Adjuvant + Fungicide	581	1, 3, 10, 30, 40, 50	104.7 (56.5–153)	111.3 (60.0–162.6)	6.86 (3.70–10.0)	1.1 (0.77–1.5)
LI 700 + Vangard	Adjuvant + Fungicide	739	1, 3, 10, 30, 40, 50	55.9*** (44.1–67.8)	59.5*** (46.9–72.1)	7.64 (6.02–9.26)	1.3 (1.0–1.6)
Activator-90 alone	Adjuvant	539	1, 3, 5, 8, 10, 12, 15	29.6 (12.0–47.2)	61.1 (24.8–97.5)	N/A	0.74 (0.47–1.0)
Liberate alone	Adjuvant	799	1, 3, 5, 8, 10, 15, 30, 50	28.5 (23.7–33.2)	28.5 (23.7–33.3)	N/A	1.4 (1.1–1.6)
Choice Weather Master alone	Adjuvant	947	1, 3, 5, 8, 10, 15, 30, 50	206 (0.0–450)	498.0 (0.0–1086.0)	N/A	0.36 (0.23–0.49)
Mustang Maxx (Positive Control) alone	Insecticide	1,209	0.1, 0.3, 0.5, 0.7, 1, 3, 10	0.59 (0.52–0.65)	N/A	0.0043 (0.0038–0.0047)	1.4 (1.6–1.2)

Values in parentheses represent the 95% confidence interval for LC_50_, LD_50_, and slope parameter estimates.

Adjuvant-pesticide combinations determined to be significantly different from the respective adjuvant alone are represented with asterisks (*P < 0.05; **P < 0.01; ***P < 0.001).

### Statistical Analysis

Raw mortality data were analyzed in R (Version 4.1.1) (R Development Core Team 2022) following the procedures in Walker et al. (2022). Briefly, the *drc* package (Ritz et al. 2015) was used to create 2-parameter log-logistic models for each treatment and the dose–response relationship for each model was evaluated. Next, for treatments demonstrating a significant dose–response relationship, the LC_50_, or median lethal concentration, expressed as *X* times the maximum application rate, and 95% confidence intervals were estimated. Finally, the *ecotox* package (Hlina et al. 2021) was used to compare LC_50_ values of the adjuvant alone and in combination with pesticides using the LC_50_ ratio test (Wheeler et al. 2006). Treatments were considered to have significantly different LC_50_ estimates if the *P*-value for the LC_50_ ratio test was less than 0.05.

### Conversion of LC_50_ (*X* Application Rate) to LD_50_ (µg/Bee)

To determine the volume of spray material applied to each bee, a group of twenty 3-day-old bees were sacrificed after cooling to −4 °C, then allowed to thaw for 30 min in the incubator. Groups of bees were weighed (Balance Mettler Toledo XS105DU), sprayed with 1-ml distilled water following the same methods used for treatments, and immediately weighed again. Five groups of 20 bees were analyzed for 3 separate brushing dates, which were used to calculate a mean for the colony sample. Three colony samples were averaged to determine average weight gain resulting from the spray by dividing the total mass gain by the number of bees sprayed (Zhu et al. 2015).

To convert adjuvant LC_50_ in *X* application rate to LC_50_ in milligrams per liter, the LC_50_ in *X* application rate was multiplied by the density (*D*) of the adjuvant as stated in the Safety Data Sheets (SDS), the maximum application rate percent concentration (in ml product per ml solution) divided by 100, and 1,000 mg/g.


$$\begin{array}{l} {\text{LC}}_{50,\text{mg/liter}}=({\text{LC}}_{50,\;\;\text{​}X\;\;\text{ application }\;\text{ rate}})\times \left(\frac{D\;\;\text{ g}}{\text{ml}}\right)\times \\ \frac{\left(\;\%\;\;\text{ Concentration }\;\text{ app }\;\text{ rate}\right)}{100}\times \frac{1,000\;\;\text{ mg}}{1\;\;\text{ g}}\times \frac{\text{1,000ml}}{\text{liter}} \end{array}$$

To convert pesticide LC_50_ in *X* application rate to LC_50_ A.I. in milligrams per liter, different conversions were needed depending on whether the pesticide was a flowable, liquid, or emulsifiable concentrate (F, L, or EC) or solid water dispersible granule (WG) formulation. For the WG pesticides, Pristine, and Vangard, the LC_50_ in *X* application rate was multiplied by the % A.I. by weight divided by 100, the maximum application rate percent concentration (in g product per ml solution) divided by 100, 1,000 mg/g, and 1,000 ml/liter.


$$\begin{array}{l} {\text{LC}}_{50,\text{mg/liter}}=({\text{LC}}_{50,\;\;\text{​}X\;\;\text{ application }\;\text{ rate}})\times \left(\frac{\;\%\;\;\text{ A.I.}}{100}\right)\times \\ \frac{\left(\;\%\;\;\text{ Concentration }\;\text{ app }\;\text{ rate}\right)}{100}\times \frac{1,000\;\;\text{ mg}}{1\;\;\text{ g}}\times \frac{\text{1,000ml}}{\text{liter}} \end{array}$$

For F, L, or EC pesticides, Altacor, Intrepid 2F, Luna Sensation, Mustang Maxx, and Tilt, the LC_50_ in X application rate was multiplied by the % A.I. by weight divided by 100, the maximum application rate percent concentration (in ml product per ml solution) divided by 100, the density (*D*) of the pesticide as stated in the SDS, 1,000 mg/g, and 1,000 mL/L.


$$\begin{array}{l} {\text{LC}}_{50,\text{mg/liter}}=({\text{LC}}_{50,\;\;\text{​}X\;\;\text{ application }\;\text{ rate}})\times \left(\frac{\;\%\;\;\text{ A.I.}}{100}\right)\\ \times \frac{\left(\;\%\;\;\text{ Concentration }\;\text{ app }\;\text{ rate}\right)}{100}\times \left(\frac{D\;\;\text{ g}}{\text{ml}}\right)\times \frac{1,000\;\;\text{ mg}}{1\;\;\text{ g}}\times \frac{\text{1,000ml}}{\text{liter}} \end{array}$$

The LC_50_ in grams was multiplied by the average mass of spray material received by each bee, determined to be 0.4088 mg/bee, to allow the determination of dose and calculation of LD_50_ in micrograms (Zhu et al. 2015).


$${\text{LD}}_{\text{50, }\mu \text{ g/bee}}=({\text{LC}}_{\text{50, }\;\text{ mg}})\times \left(\frac{\text{0.4088 }\;\text{ mg}}{\text{bee}}\right)/1,000$$

## Results

None of the individual fungicide or insecticide formulations demonstrated a dose–response relationship significantly different from zero over the 4–6 concentrations that were tested, nor did the adjuvants Attach, PHT Latron B-1956, or Nu-Film P, as determined by the noEffect test in the drc package in R (*P* > 0.05) (Ritz et al. 2015), by the LC_20_ value exceeding the maximum concentration tested, or by having a dose–response relationship for which the LC_20_ cannot be determined (Table 3; Supplementary Table S3). Nine of the 12 adjuvants generated significant dose–response relationships, determined by the noEffect test in the drc package in R (*P* < 0.05) and by having an LC_20_ value less than the maximum concentration tested. These included Kinetic (χ^2^ = 782, df = 1, 1120; *P* < 0.001), Induce (χ^2^ = 253, df = 1, 402; *P* < 0.001), Cohere (χ^2^ = 147, df = 1, 463; *P* < 0.001), Surf-90 (χ^2^ = 526, df = 1, 1263; *P* < 0.001), Dyne-Amic (χ^2^ = 531, df = 1, 1995; *P* < 0.001), LI 700 (χ^2^ = 159, df = 1, 1475; *P* < 0.001), Activator-90 (χ^2^ = 35.4, df = 1, 539; *P* < 0.001), Liberate (χ^2^ = 222, df = 1, 799; *P* < 0.001), and Choice Weather Master (χ^2^ = 31.5, df = 1, 947; *P* < 0.001), both when applied alone and in all tested combinations with pesticides (Supplementary Table S3), excluding the combination of Dyne-Amic adjuvant and Tilt fungicide, for which LC_50_ values and LD_50_ values (Table 4) were estimated. The number of replicates and the respective concentrations tested varied between treatments as additional concentrations were added after initial bioassays to increase precision for LD_50_ and LC_50_ estimates.

**Fig. 2.**
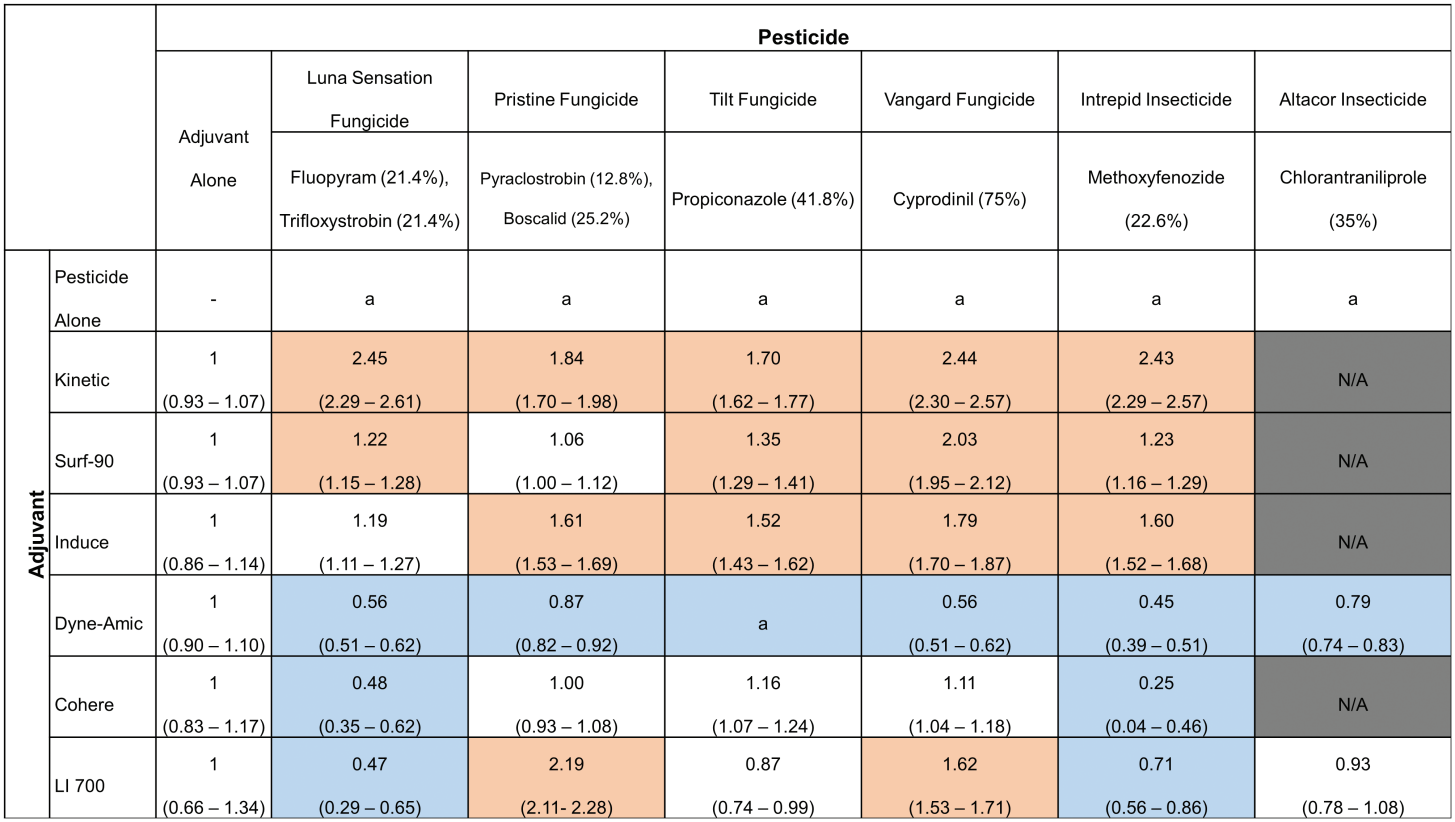
The ratio of LC_50_ estimates of adjuvant-pesticide combinations compared to the adjuvant alone when three-day-old adult worker honey bees were treated alone and in combination with a single fungicide or insecticide. Ratios were determined by dividing the LC_50_ of the adjuvant alone by the LC_50_ of the adjuvant-pesticide combination. Parentheses indicate ratio 95% confidence intervals. “N/A” indicates combinations that were not tested. The letter “a” indicates that there was no significant dose-response relationship for the given treatment. Adjuvant-pesticide combinations are determined to be significantly different from adjuvants alone when *P* < 0.05, with shaded cells indicating a significant increase in toxicity (ratio > 1) or a significant decrease in toxicity (ratio < 1) in the adjuvant-pesticide combinations in comparison to the adjuvant alone.

Adjuvant-pesticide combinations were significantly more toxic than the adjuvant alone, determined by the LC_50_ ratio test in the ecotox package in R (*P* < 0.05) (Wheeler et al. 2006, Hlina et al. 2021), for the adjuvants Kinetic when applied with Luna Sensation (*Z* = 4.80, *n* = 1120, 565; *P* < 0.001), Pristine (*Z* = 3.72, *n* = 1120, 485; *P* < 0.001), Tilt (*Z* = 5.84, *n* = 1120, 404; *P* < 0.001), Vangard (*Z* = 5.76, *n* = 1120, 410; *P* < 0.001), and Intrepid (*Z* = 5.44, *n* = 1120, 365; *P* < 0.001); Surf-90 when applied with Luna Sensation (*Z* = 2.6, *n* = 1263, 471; *P* < 0.01), Tilt (*Z* = 4.07, *n* = 1263, 495; *P* < 0.001), Vangard (*Z* = 6.87, *n* = 1263, 551; *P* < 0.001), and Intrepid (*Z* = 2.69, *n* = 1263, 401; *P* < 0.01); Induce when applied with Pristine (*Z* = 5.02, *n* = 402, 347; *P* < 0.001), Tilt (*Z* = 3.87, *n* = 402, 479; *P* < 0.001), Vangard (*Z* = 6.01, *n* = 402, 469; *P* < 0.001), and Intrepid (*Z* = 4.90, *n* = 1475, 581; *P* < 0.001); and LI 700 when applied with Pristine (*Z* = 8.01, *n* = 402, 347; *P* < 0.001) and Vangard (*Z* = 4.46, *n* = 1475, 520; *P* < 0.001) (Fig. 2; Supplementary Table S2).

Adjuvant-pesticide combinations were significantly less toxic than the adjuvant applied alone, determined by the LC_50_ ratio test in the ecotox package in R (*P* < 0.05) (Wheeler et al. 2006, Hlina et al. 2021), for Dyne-Amic combinations with Luna Sensation (*Z* = 8.46, *n* = 1995, 820; *P* < 0.001), Pristine (*Z* = 2.44, *n* = 1995, 958; *P* < 0.05), Tilt (*Z* = 11.3, *n* = 1995, 996; *P* < 0.001), Vangard (*Z* = 8.46, *n* = 1995, 1019; *P* < 0.001), Altacor (*Z* = 2.44, *n* = 1995, 763; *P* < 0.001), and Intrepid (*Z* = 10.6, *n* = 1995, 600; *P* < 0.001); Cohere combinations with Luna Sensation (*Z* = 4.24, *n* = 463, 528; *P* < 0.001) and Intrepid (*Z* = 5.57, *n* = 463, 404; *P* < 0.001); and LI 700 combinations with Luna Sensation (*Z* = 3.62, *n* = 1475, 522; *P* < 0.001) and Intrepid (*Z* = 1.98, *n* = 1475, 440; *P* < 0.05) (Fig. 2; Supplementary Table S2).

Adjuvant toxicity was measured in multiples of the maximum labeled application rate. However, adjuvant application rates vary between products, with maximum labeled rates varying from 1,156 (8 fl oz. per 100 gallons) to 5,719 mg/liter (80 fl oz. per 100 gallons). Higher maximum application rates for adjuvant products were found to be correlated with higher observed bee toxicity when measured in units of multiples of the maximum application rate (Spearman’s correlation, *R* = −0.640, df = 10; *P* = 0.0249).

## Discussion

**Fig. 3.**
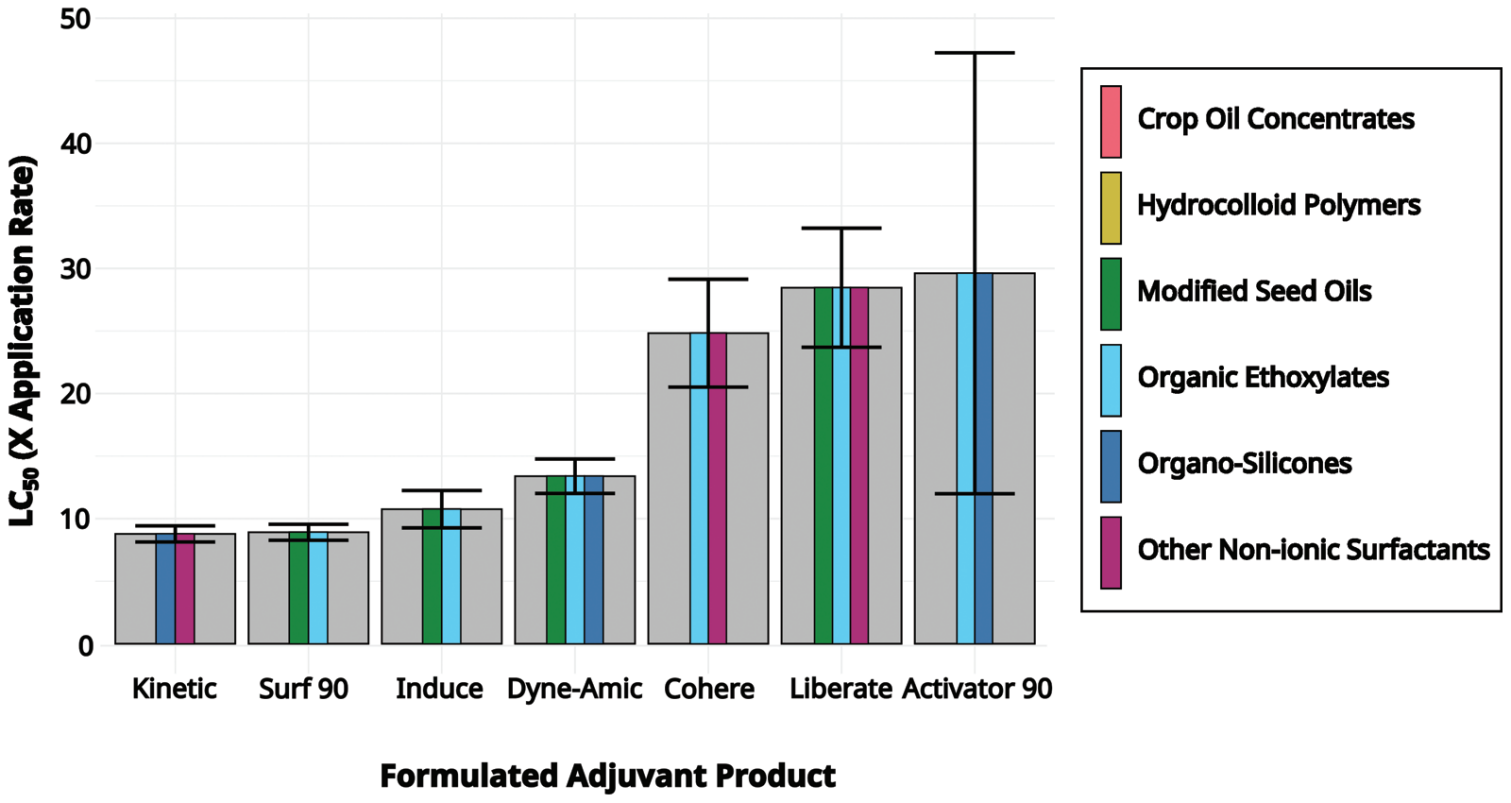
LC_50_ estimates for the 7 of the 12 adjuvants tested that demonstrated toxicity at near application rates (less than 30 times the maximum labeled application rate). LC_50_ is expressed as multiples of the maximum application rate listed on the label. The shade of vertical bars indicate the class or classes of principal functioning agent present based on information from the label. Error bars indicate 95% confidence intervals for LC_50_ estimates.

Seven of the 12 adjuvants alone demonstrated acute toxicity to 3-day-old adult worker honey bees at concentrations near the maximum field application rate (less than 30 multiples of the maximum labeled rate), including Kinetic, Surf-90, Induce, Dyne-Amic, Cohere, Liberate, and Activator-90. There does not appear to be a relationship between the toxicity of formulated adjuvants and the class or classes of principal functioning agents these products contain (Fig. 3). Drawing conclusions about the relative hazard of different classes of adjuvants is stymied by the fact that many adjuvants are mixtures of multiple principal functioning agents and there is a lack of detailed information regarding the identity of these constituents. Many adjuvant labels list principal functioning agents with ambiguous descriptions such as “methyl esters of fatty acids,” “nonionic surfactants,” or “polymers” with proprietary molecular weights. These broad categories could include a wide range of possible compounds, each with different physical and chemical properties. The precise identity and relative proportions of each specific principal functioning agent are considered proprietary information and are generally not listed on the label. This lack of transparency in the adjuvant constituents limits research on the effect of specific components in formulated adjuvants on honey bees.

The mechanism or mode of action for surfactant toxicity to honey bees or other arthropods is currently not well understood. Most surfactants have amphiphilic properties similar to soaps, with a hydrophobic group, usually a long-chain hydrocarbon, and hydrophilic group made up of an ionic or highly polar group (Cirelli et al. 2010, Castro et al. 2014). This leads us to hypothesize that adjuvants have a similar mode of action to insecticidal soaps, which are made up of a dissolved cation and fatty acid. (Ralston et al. 1941, Puritch 1975, Parry and Rose 1983, Baldwin et al. 2008). Soaps can disrupt the insect cuticle and break down cell membranes to cause rapid death in insects and mites, as well as reduce water surface tension to penetrate spiracles and cause drowning (Ware and Whitacre 2004, Yu 2008). Fatty acids of insecticidal soaps exert hemolytic action, inhibit proteolytic enzymes, and cause immediate paralysis in aphids (Siegler and Popenoe 1925). It is likely that spiracular penetration is driving toxicity (Fulton 1930, Dills and Menusan 1935, Richards and Weygandt 1945, Szumlas 2002, Abbasi et al. 2013), as tarsal contact does not cause mortality and the tracheal system of cockroaches treated with dyed soap solutions show evidence of soap solution penetration (Baldwin et al. 2008). Organo-silicones have demonstrated sufficient reduction of surface tension to cause water infiltration into spiracles of insects and other arthropods, causing asphyxiation (Dentener and Peetz 1992, Stevens 1993). However, some organo-silicones may work through other mechanisms, as a cyclic siloxane, octamethylcyclotetrasiloxane, has shown reproductive toxicity and endocrine effects in mammals (Lassen et al. 2005, Quinn et al. 2007).

Adjuvant-pesticide combinations, including combinations containing the adjuvants Kinetic, Induce, Surf-90, and LI 700, were found to be more toxic in combination with pesticides than alone. This increased toxicity could be caused by synergism between the adjuvant and the pesticide active ingredient or the inert ingredients in pesticide formulations. This increased toxicity may be the result of increased spiracular or cuticular penetration as a result of the surfactant activity of the adjuvant, as observed in studies with similar adjuvants containing oil-based, nonionic, or organo-silicone principal functioning agents (Olson and O’Brien 1963, Lewis 1980, Dentener and Peetz 1992, Fanning et al. 2018). Increased toxicity was observed for all tested pesticides in combination with the adjuvant Kinetic, 4 of the 5 tested pesticides in combination with Induce or Surf-90, and 2 of the 5 tested pesticides in combination with LI 700. However, some adjuvant-pesticide combinations, including the adjuvants Cohere, Dyne-Amic, and LI 700, were found to be less toxic than the adjuvant alone—indicating potential antagonism through an unknown mechanism. Decreased toxicity, relative to the adjuvant alone, was observed in combinations of all 6 of the tested pesticides mixed with the adjuvant Dyne-Amic and 2 of the 5 tested pesticides in combinations with Cohere or LI 700. Antagonism of insecticide toxicity when combined with adjuvants has also been observed in aphids (Radwan et al. 1982). Adjuvant-herbicide mixtures have also shown antagonism, which may be due to the increased pH of the spray solution, which can cause the appearance of precipitates (Nalewaja et al. 1991, Kirkwood 1993). Some organic ethoxylate surfactants have been shown to reduce uptake of the herbicide active ingredient glyphosate (van Toor et al. 1994). However, there were no trends in synergism or antagonism for pesticides included in tank mix combinations, which suggests that these effects are adjuvant dependent in all cases except LI 700, which showed either synergistic or antagonistic effects depending on the pesticide.

The toxicity of tank mixtures to honey bees may be underestimated by this study. Three-day-old adult worker honey bees were used following common practice for honey bee acute toxicity testing that requires all bees to be the same age (US EPA 2012), but older forager bees are more likely to be exposed to direct spray application and are more sensitive to pesticides than in-hive bees (Wahl and Ulm 1983, Vance et al. 2009, Tosi and Nieh 2019). Using 3-day-old bees for laboratory testing limits the high control mortality observed when older foragers are confined to cages. Mortality was used as the endpoint for acute toxicity testing in this study, but sublethal effects can be caused by pesticides (Yao et al. 2018, Prado et al. 2019), as can adjuvants (Ciarlo et al. 2012), and adjuvant-pesticide combinations (Artz and Pitts-Singer 2015).

More work is needed to evaluate the sublethal and the colony-level effects of formulated adjuvants alone and in real-world combinations with pesticides. Additionally, only one adjuvant and one pesticide were tested in combination in this study, while real tank mixes may contain more than two products. There is the potential for more complex mixtures of pesticides and adjuvants to interact synergistically and increase the hazard to foraging honey bees (Tosi and Nieh 2019). While combinations of individual fungicide-adjuvant tank mix combinations showed no significant effect on queen development or survival (Johnson and Percel 2013), the combination of an insecticide, fungicide, and adjuvant did affect queen development and survival (Ricke et al. 2021). Synergistic interactions can also occur between pesticides and other bee health factors, such as poor nutrition (Tosi et al. 2017) or diseases (Alaux et al. 2010). Additionally, adjuvants can increase the pathogenicity of bee viruses and mortality in larvae (Fine et al. 2017, Chen et al. 2018). The 48-hour acute toxicity testing on adult workers does not capture sublethal effects and colony-level effects that may be caused by adjuvants and adjuvant-pesticide tank mix combinations.

This study used a Potter Spray Tower to mimic adjuvant exposure honey bees receive when directly sprayed with agrochemicals while foraging. Direct spray is thought to be the most likely route of exposure for adjuvants to honey bees (Slade 2020). Adult worker honey bees could also be exposed to adjuvants through other routes, including affected water sources, pollen, nectar, and residual concentrations in hive products. Some adjuvants can have a half-life of up to 6 months in water bodies where they can cause a reduction in surface tension, leading to honey bee drowning, at concentrations as low as 25 ppm (Moffett and Morton 1973, 1975). Residue studies of hive products demonstrated that organo-silicone adjuvants can be detected in pollen (Chen and Mullin 2013), though levels found in almond pollen are unlikely to cause honey bee mortality (Collins and Jackson 2022). This study did not investigate risks associated with oral or chronic exposure and may not fully capture the effects of adjuvants on honey bees.

What defines “near application rate” is a subject of debate and depends on the safety buffer built into application rates in relation to beneficial insect toxicity. However, the maximum application rate listed on the label is not always the worst-case scenario. Higher application rates could result from either mixing errors or increased adjuvant use rates specified for aerial applications. While Kinetic and Surf-90 have the same labeled rate for aerial applications, the maximum labeled rate is up to 100 times higher for Nu-Film P, 100 times higher for Attach, 60 times higher for Dyne-Amic, and 6 times higher for PHT Latron B-1956. Estimated bee exposure outside of the orchard targeted for aerial or ground applications could be refined using the AgDRIFT spray drift assessment tool, which can estimate the point deposition of a tank mix spray at varying distances from the edges of almond orchards (Teske et al. 2002, US EPA 2002). The results presented here are based on maximum ground application rates. However, pesticide use reporting data from California show that most pesticides and adjuvant applications are generally at or below the maximum recommended application rate (Supplementary Figs. S1 and S2) (State of California – California Pesticide Information Portal 2023). Adjuvants have a much wider range of application rates when compared to pesticides and most applications are well below the maximum labeled rate.

Performing a Spearman’s correlation on the California Pesticide Usage Data for adjuvants, fungicides, and insecticides applied in almond orchards during bloom indicates that insecticide usage has been decreasing over recent years (*R* = −0.62, df = 8; *P* = 0.043) (Supplementary Fig. S3). However, fungicide (*R* = −0.50, df = 8; *P* = 0.12) and adjuvant usage (*R* = −0.37, df = 8; *P* = 0.26) do not show a significant trend using Spearman’s correlation and have remained relatively consistent (Supplementary Figs. S4 and S5), highlighting the need to focus on adjuvant and adjuvant-pesticide tank mix toxicity to honey bees. The 12 tested adjuvants were applied to approximately 144,000 hectares of almond orchards in 2020, which corresponds to 28.7% of all California almond-bearing land area. We consider adjuvants to be acutely toxic to bees at near application rates when the LC_50_ estimate is less than 30 multiples of the maximum application rate due to the toxicity occurring below 50% mortality and safety factors that include interactions between products in tank mixes and sublethal effects to honey bees. The 7 adjuvants with LC_50_ estimates determined to be near application rates were applied to 117,000 hectares of almond orchards in 2020, which corresponds to 23.2% of all almond land area (State of California – California Pesticide Information Portal 2023). These 7 adjuvants with respective toxicities at near application rates would affect an estimated 576,000 managed honey bee colonies at a density of 2 colonies per acre (Goodrich et al. 2021), in addition to non-managed beneficial organisms. It is likely that these adjuvants are causing adverse effects on pollinating honey bees that can be linked to the reported beekeeper colony losses during and after almond bloom.

In communicating risk of adjuvants to pesticide applicators, it is important to understand the misperceptions held regarding adjuvants, as their constituents are on the EPA “inert ingredient” list. In a risk communication strategy that seeks to limit the number of uses and application rates of adjuvants used on bee-pollinated crops, it will be important for the risk communicator to first dispel misconceptions about how the EPA defines “inert ingredients” before effectively filling knowledge gaps about the risks of adjuvants (Bostrom et al. 1992, Maharik and Fischhoff 1992, Atman et al. 1994, Morgan et al. 2002).

Based on this research, we recommend that pesticide applicators working in almonds and other bee-pollinated crops use adjuvants only when they are specified on the pesticide label, which indicates that the pesticide manufacturer recommends adding an adjuvant to enhance efficacy but does not necessarily mean that these adjuvant-pesticide combinations were tested for toxicity. If adjuvants must be used, then bee exposure can be minimized by applying pesticides at night or other times of the day when bees are not actively foraging and by using adjuvants at rates lower than the maximum application rate, as higher application rates are correlated with higher honey bee toxicity. In particular, adjuvants demonstrating bee toxicity at near-field application rates should be avoided, including Kinetic, Surf-90, Induce, Dyne-Amic, Cohere, Liberate, and Activator-90. Beekeepers should communicate with pollination clients about the pest management strategies that will be used on crops, particularly the components of the tank mixtures and the time of day at which they will be applied. Communication between parties can improve willingness to cooperate and build trust (Loomis 1959, Cooper et al. 1989, Sally 1995, Hardman 2009).

## Supplementary Material

toad161_suppl_Supplementary_Figures

toad161_suppl_Supplementary_Tables

## Data Availability

The datasets generated by the current study are available in Supplementary Materials.
