# Supplementary material for: Toxicity of spray adjuvants and tank mix combinations used in almond orchards to adult honey bees (Apis mellifera)
Source: J Econ Entomol. 2023 Sep 1;116(5):1467–80. doi: 10.1093/jee/toad161 (PMC10564267; doi:10.1093/jee/toad161)
Supplement: toad161_suppl_Supplementary_Figures [file toad161_suppl_supplementary_figures.pdf]

Article Title: Toxicity of Spray Adjuvants and Tank Mix Combinations Used in Almond Orchards to Adult Honey Bees

Journal Name: Journal of Economic Entomology

Author Names: Brandon Shannon*1, Emily Walker*1, Reed Johnson1

*-both authors contributed equally to this work;

1-The Ohio State University, Dept. of Entomology, Wooster, OH, USA

Corresponding Author Email: Shannon.325@OSU.edu

Supplementary Materials Figures S1 – S5

List of Supplementary Material Figures

Figure S1 Violin plots of usage data of concentrations for spray adjuvants. Rates of application are represented on the y-axis in log scale as a proportion of the maximum labelled application rate. The frequency of each rate is represented by the width of the violin, with a wider violin representing a higher probability of that rate being applied. The horizontal line represents the maximum rate recommended on the label. Data was summarized from pesticide usage reporting during bloom (Feb 15 – Mar 15) in the California Pesticide Information Portal (State of California - California Pesticide Information Portal 2023) SM–3

Figure S2 Violin plots of usage data of concentrations for pesticides. Rates of application are represented on the y-axis in log scale as a proportion of the maximum labelled application rate. The frequency of each rate is represented by the width of the violin, with a wider violin representing a higher probability of that rate being applied. The horizontal line represents the maximum rate recommended on the label. Data was summarized from pesticide usage reporting during bloom (Feb 15 – Mar 15) in the California Pesticide Information Portal (State of California - California Pesticide Information Portal 2023) SM–4

Figure S3 Spearman's correlation between year and proportion of California almond acres treated with insecticides. Data was summarized from pesticide usage reporting during bloom (Feb 15 – Mar 15) from 2010 to 2020 in the California Pesticide Information Portal (State of California - California Pesticide Information Portal 2023). Insecticides show a statistically significant decrease in usage over this time period ($P < 0.05$) SM–5

Figure S4 Spearman's correlation between year and proportion of California almond acres treated with fungicides. Data was summarized from pesticide usage reporting during bloom (Feb 15 – Mar 15) from 2010 to 2020 in the California Pesticide Information Portal (State of California - California Pesticide Information Portal 2023). Fungicides do not show a statistically significant decrease in usage over this time period ($P < 0.05$) SM–6

Figure S5 Spearman's correlation between year and proportion of California almond acres treated with adjuvants. Data was summarized from pesticide usage reporting during bloom (Feb 15 – Mar 15) from 2010 to 2020 in the California Pesticide Information Portal (State of California - California Pesticide Information Portal 2023). Adjuvants do not show a statistically significant decrease in usage over this time period ($P < 0.05$) SM–7

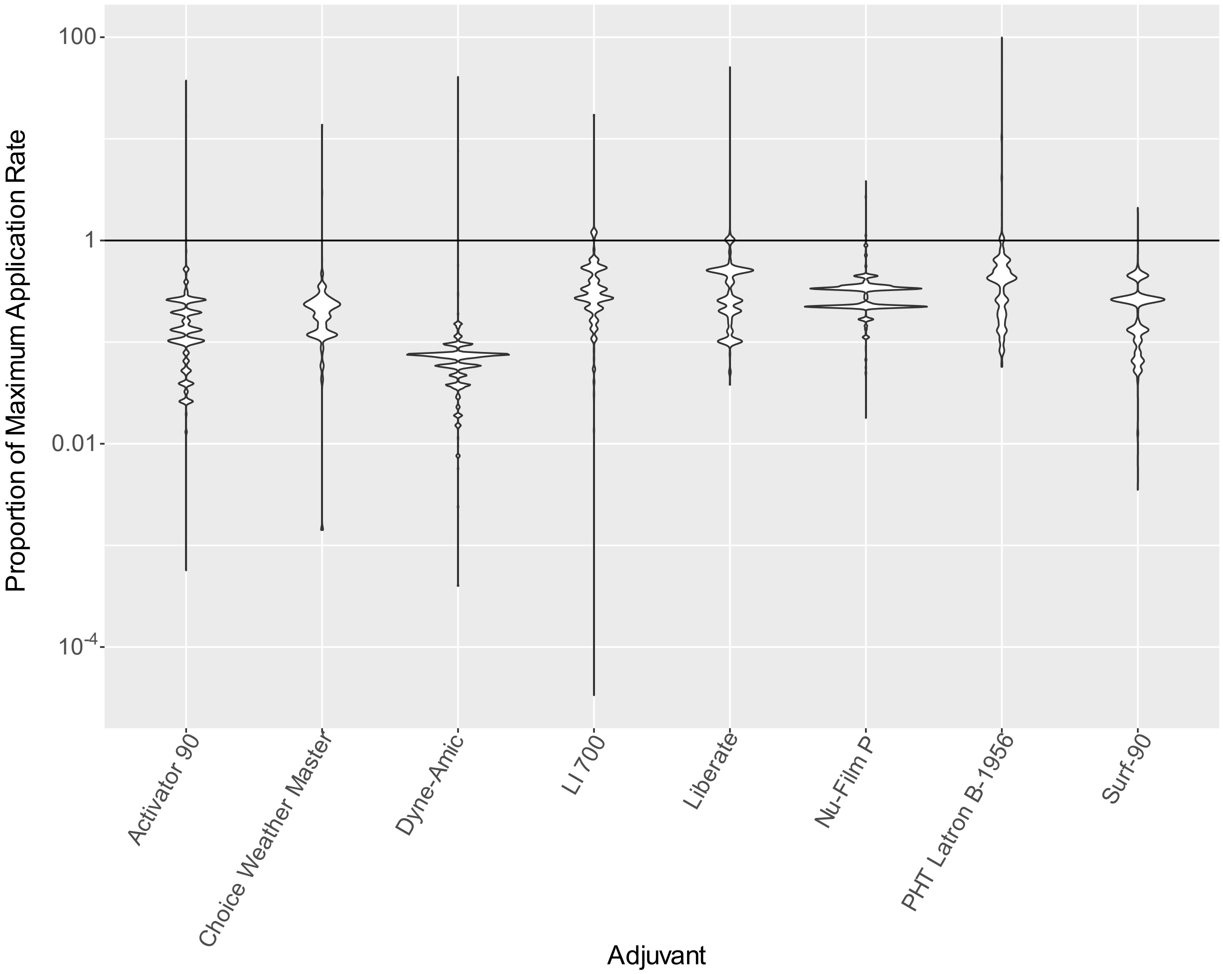

Figure S1 Violin plots of usage data of concentrations for spray adjuvants. Rates of application are represented on the y-axis in log scale as a proportion of the maximum labelled application rate. The frequency of each rate is represented by the width of the violin, with a wider violin representing a higher probability of that rate being applied. The horizontal line represents the maximum rate recommended on the label. Data was summarized from pesticide usage reporting during bloom (Feb 15 – Mar 15) in the California Pesticide Information Portal (State of California - California Pesticide Information Portal 2023)

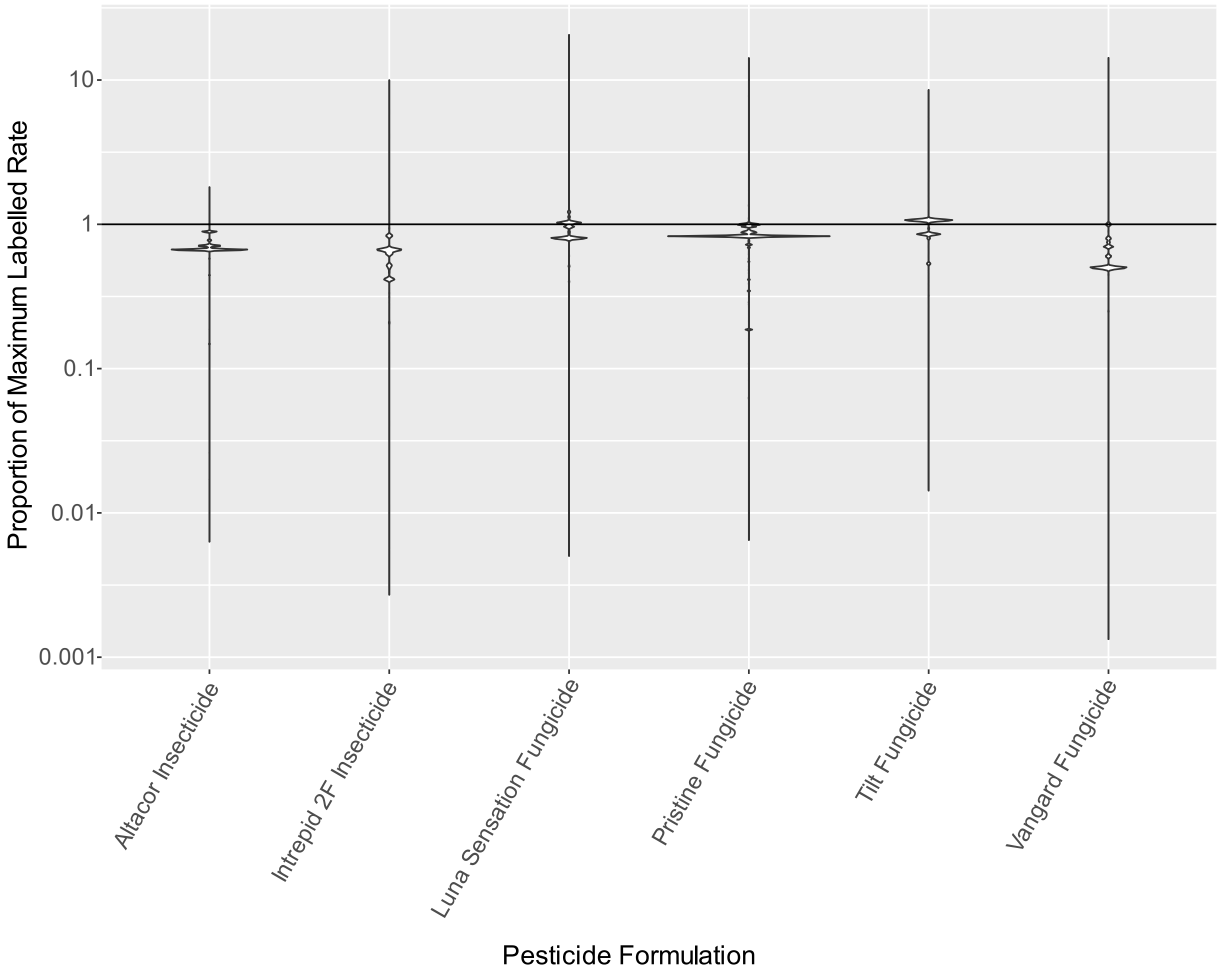

Figure S2 Violin plots of usage data of concentrations for pesticides. Rates of application are represented on the y-axis in log scale as a proportion of the maximum labelled application rate. The frequency of each rate is represented by the width of the violin, with a wider violin representing a higher probability of that rate being applied. The horizontal line represents the maximum rate recommended on the label. Data was summarized from pesticide usage reporting during bloom (Feb 15 – Mar 15) in the California Pesticide Information Portal (State of California - California Pesticide Information Portal 2023)

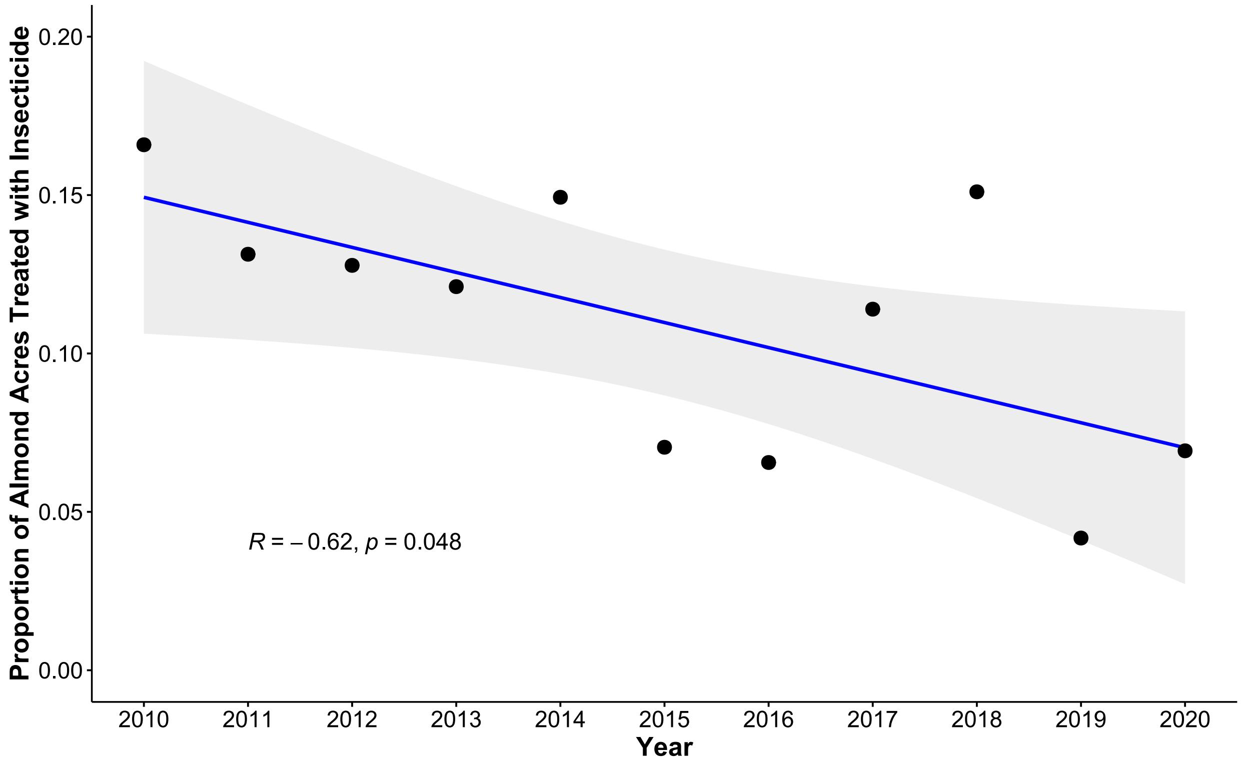

Figure S3 Spearman's correlation between year and proportion of California almond acres treated with insecticides. Data was summarized from pesticide usage reporting during bloom (Feb 15 – Mar 15) from 2010 to 2020 in the California Pesticide Information Portal (State of California - California Pesticide Information Portal 2023). Insecticides show a statistically significant decrease in usage over this time period ($P < 0.05$)

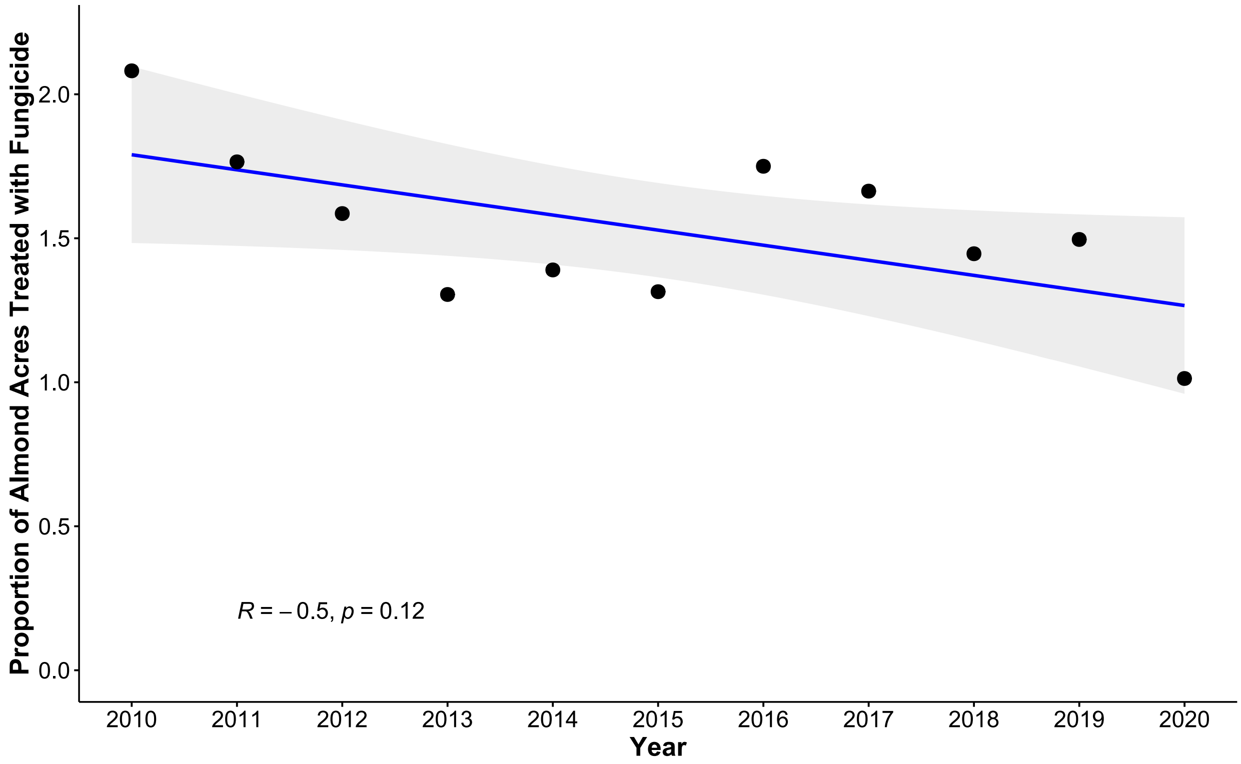

Figure S4 Spearman's correlation between year and proportion of California almond acres treated with fungicides. Data was summarized from pesticide usage reporting during bloom (Feb 15 – Mar 15) from 2010 to 2020 in the California Pesticide Information Portal (State of California - California Pesticide Information Portal 2023). Fungicides do not show a statistically significant decrease in usage over this time period ($P < 0.05$)

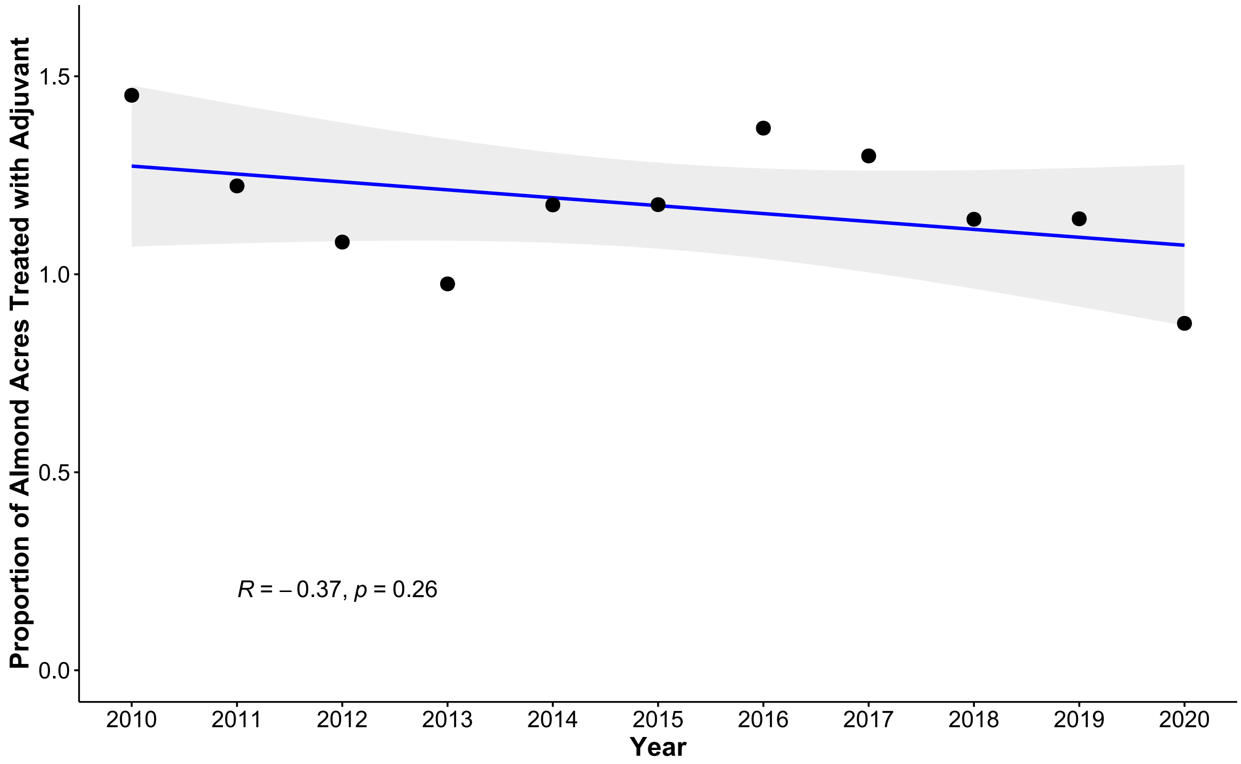

Figure S5 Spearman's correlation between year and proportion of California almond acres treated with adjuvants. Data was summarized from pesticide usage reporting during bloom (Feb 15 – Mar 15) from 2010 to 2020 in the California Pesticide Information Portal (State of California - California Pesticide Information Portal 2023). Adjuvants do not show a statistically significant decrease in usage over this time period ($P < 0.05$)
